# Using appropriate pre-pregnancy body mass index cut points for obesity in the Chinese population: a retrospective cohort study

**DOI:** 10.1186/s12958-018-0397-z

**Published:** 2018-08-10

**Authors:** Yanxin Wu, Wai-Kit Ming, Dongyu Wang, Haitian Chen, Zhuyu Li, Zilian Wang

**Affiliations:** Department of Obstetrics and Gynecology, The First Affiliated Hospital of Sun Yat-sen University, No. 58 Zhongshan Road 2, Guangzhou, 510000 P. R. China

**Keywords:** Obesity, Pregnancy, Body mass index, Outcome

## Abstract

**Background:**

Appropriate classification of obesity is vital for risk assessment and complication prevention during pregnancy. We aimed to explore which pre-pregnancy BMI cut-offs of obesity, either BMI ≥ 25 kg/m^2^ as recommended by the WHO for Asians or BMI ≥ 28 kg/m^2^ as suggested by the Working Group on Obesity in China (WGOC), best predicts the risk of adverse maternal and perinatal outcomes.

**Methods:**

We retrospectively reviewed 11,494 medical records for live singleton deliveries in a tertiary center in Guangzhou, China, between January 2013 and December 2016. The primary outcomes included maternal obesity prevalence, adverse maternal and perinatal outcomes. Data were analyzed using the Chi-square test, logistic regression, and diagnostics tests.

**Results:**

Among the study population, 824 (7.2%) were obese according to the WHO criteria for Asian populations, and this would be reduced to 198 (1.7%) based on the criteria of WGOC. Obesity-related adverse maternal and perinatal outcomes were gestational diabetes mellitus, preeclampsia, cesarean section, and large for gestational age (*P* < 0.05). Compared to the WGOC criterion, the WHO for Asians criterion had a higher Youden index in our assessment of its predictive value in identifying risk of obesity-related adverse outcomes for Chinese pregnant women. Women in the BMI range of 25 to 28 kg/m^2^ are at high risks for adverse maternal and perinatal outcomes, which were similar to women with BMI ≥ 28 kg/m^2^.

**Conclusions:**

A lower pre-pregnancy BMI cutoff at 25 kg/m^2^ for defining obesity may be appropriate for pregnant women in South China. If WGOC standards are applied to pregnant Chinese populations, a significant proportion of at-risk patients may be missed.

## Background

The prevalence of obesity is on the rise throughout China and the rest of the world in recent decades, including women of childbearing age [1, 2]. Greater adiposity in pregnancy is associated with increased risk of gestational diabetes, hypertensive disorders of pregnancy, higher birth weight, preterm delivery, large for gestational age, and cesarean section (C/S) [3–6]. Therefore, to ensure these women can receive medical advice and closer monitoring, many national and international antenatal guidelines recommend an early diagnosis of overweight and obesity in women of childbearing age [7, 8].

According to the World Health Organization (WHO), the cut-offs of BMI for defining overweight and obesity for Caucasian populations are 25 and 30 kg/m^2^ [9]. Previous studies have shown that the Chinese, as well as other Asian populations, have a lower BMI but a higher percentage of body fat than Caucasians of similar age and gender [10, 11]. As such, the BMI criterion for Asian populations should be lowered so as to better suit the characteristics of this racial group. For Asian populations, the expert group of the WHO defines BMIs of 23.0–24.9 kg/m^2^ as overweight and ≥ 25.0 kg/m^2^ as obesity [12]. This expert group has labeled these recommendations as temporary and stated that these need to be further validated by additional epidemiological research, owing to limited information available about Asian populations [13]. BMI cut-off points of 24.1–27.9 kg/m^2^ for overweight and ≥ 28.0 kg/m^2^ for obesity have been proposed by the Working Group on Obesity in China (WGOC) and the International Life Sciences Institute Focal Point in China [14, 15]. The proposal was primarily based on a large, national population (20- to 70-years-old), cross-sectional study of anthropometric indices and cardiovascular risk factors. Optimal classification of obesity is vital for risk assessment and weight management, for both the individual and health professionals. However, it is unclear whether these recommendations can also adequately reflect the risk of adverse maternal and perinatal outcomes among pregnant women.

The objective of this study was to investigate the application of obesity criterion of BMI recommended by the WHO for Asians and WGOC in pregnant Chinese women to identify which recommendation best identifies those at risk of adverse maternal and perinatal outcomes.

## Methods

### Study population and clinical data

This study is a retrospective cohort study. We reviewed the medical records of ethnically Chinese women with singleton pregnancy and a live delivery at the First Affiliated Hospital of Sun Yat-sen University, Guangzhou, China, between January 2013 and December 2016. Inclusion criteria were as follows: gave birth at 28 or more completed weeks of gestation; and complete antenatal and birth data. Exclusion criteria were as follows: preexisting diabetes and hypertension. A total of 11,494 women were included, among which 9,178 (79.9%) were nulliparous. The study was approved by an ethics committee of The First Affiliated Hospital of Sun Yat-sen University.

Demographic data were collected for each participant and included maternal age (years), clinical history, pre-pregnancy weight (self-reported or any measured weights during the 1 year before pregnancy), height, and prenatal care (date of prenatal visit and complications of pregnancy). Pre-pregnancy BMI was calculated as weight in kilograms (kg) before pregnancy, divided by height in meters squared (m^2^). Maternal obesity was defined using the WHO classification for Asian populations (BMI ≥ 25kg/m^2^) and WGOC (BMI ≥ 28kg/m^2^). All possible thresholds were defined for BMIs between 19 and 28kg/m^2^ in three unit increments. Adverse obstetrics outcomes were compared in the classification among the study population.

Maternal and perianaloutcomes included prevalence of C/S,operative vaginal delivery (OVD), preeclampsia (gestational systolic BP ≥ 140mmHg ordiastolic BP ≥ 90mmHg [at least two readings, 4h apart], with 1+ of proteinuria or more on dipstick), gestational diabetes (according to the criteria established by the American Diabetes Association) [16], and postpartum hemorrhage. Outcomes among neonates included preterm birth (delivered <37^+0^ weeks gestation), shoulder dystocia, large for gestational age (LGA), small for gestational age (SGA), a low Apgar score (1 or 5-min Apgar score less than 7), and admission to the neonatal intensive care unit (NICU). LGA and SGAwere defined as birth weight above or below the 90^th^ and 10^th^ centiles of the local scale, respectively, after adjusting for gender and gestational age [17].

### Statistical analysis

All statistical analyses were performed using the Statistical Package for Social Science version 21.0 (SPSS, Inc., Chicago, IL, USA) and R 3.3.1 (R Project for Statistical Computing., Vienna, Austria). Data are expressed as mean ±standard deviations (SD) for normally distributed variables, median with interquartile range for skewed data, and as frequencies for categorical variables. Differences between groups were assessed using the Mann-Whitney U-test, Kruskal-Wallis test or Chi-squared test, as appropriate. The BMI group of 19~22 kg/m^2^ was used as the reference or comparison group. Maternal age and previous C/S were considered as confounding factors in the determination of all adjusted odds ratios in relation to BMI level. The risks of maternal and perinatal complications were presented as adjusted odds ratio with 95% confidence interval (95% CI) after adjusting for the confounding factors. We adopted the Chi-square test for trend to investigate whether the prevalence of adverse obstetric outcomes in different BMI groups manifested a linear trend. Moreover, bootstrap resampling was performed 1000 times to calculate the 95%CI of the Youden indexes criteria and the 95%CI of the difference of Youden indexes between the two criteria, thereby revealing their predictive effect on adverse maternal and perinatal outcomes. P-value less than 0.05 was defined as of statistical significance. If the 95%CI of the difference of Youden indexes between the two criteria did not cover a zero value, it was indicated that the Youden indexes of the two criteria were at statistically different levels.

## Results

### Obesity prevalence

**Fig. 1:**
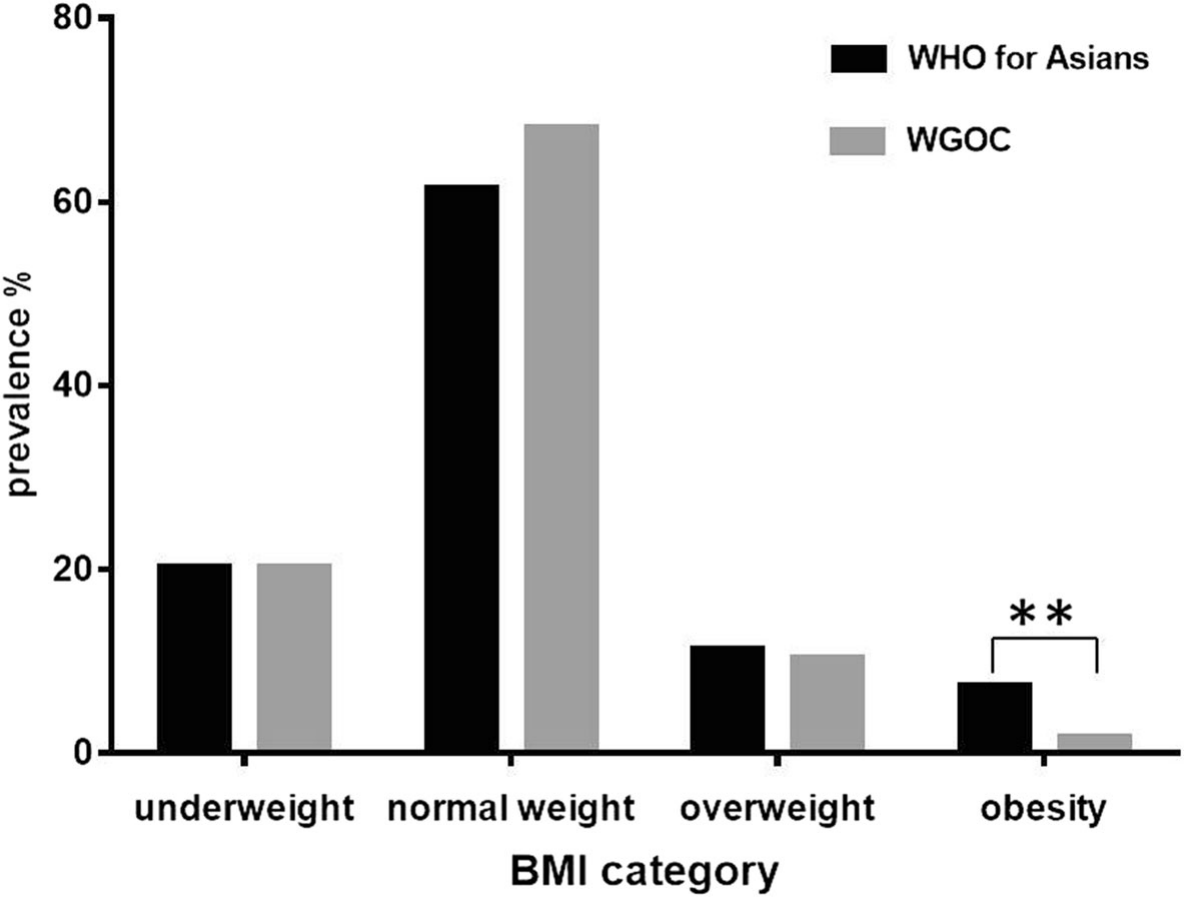
Obesity defined by WHO for Asian population and WGOC specific BMI cut-offs among women from the First Affiliated Hospital of Sun Yat-sen University, Guangzhou, China. BMI (kg/m^2^) < 18.5 = Underweight. BMI for Asian population (kg/m^2^): 18.5-22.9 = normal weight, 23.0-24.9 = overweight, ≥ 25 = obesity. BMI for WGOC (kg/m^2^): 18.5-23.9 = normal weight, 24.0-27.9 = overweight, ≥ 28 = obesity. ** *P*<0.001

The prevalence of obesity was 7.2% (95% CI, 6.7~7.7) according to the WHO criteria for Asian populations (BMI ≥ 25kg/m^2^); this proportion decreased to 1.7% (95% CI, 1.5~2.0) when using the Chinese-specific threshold (BMI ≥ 28kg/m^2^) (Fig. 1). The characteristics of the women are listed in Table 1. Mothers with obesity were more likely to be older, have a previous cesarean delivery, shorter gestational week, and higher birth weight of their offspring (*p* < 0.001).

**Table 1:** Baseline characteristics of this study’s population^a^

	Prepregnancy BMI category (kg/m^2^)					
	BMI<19 (*n*= 3135)	19≤BMI<22 (*n*= 5113)	22≤BMI<25 (*n*= 2422)	25≤BMI<28 (*n*= 626)	BMI≥28 (*n*= 198)	*P*
Maternal age (years)	29.7 ± 4.0	31.4 ± 4.2	32.5 ± 4.3	33.0 ± 4.2	32.5 ± 4.4	<0.001
Nulliparity	2482 (79.2)	4093 (80.1)	1943 (80.2)	504 (80.5)	156 (78.8)	0.82
Previous C/S	276 (8.8)	711 (13.9)	448 (18.5)	147 (23.5)	41 (20.7)	<0.001
Gestational week	38.9 ± 1.56	38.9 ± 1.5	38.7 ± 1.6	38.6 ± 1.7	38.4 ± 1.9	<0.001
Birth weight (grams)	3060.7 ± 425.6	3159.9 ± 437.2	3210.6 ± 477.7	3263.4 ± 486.1	3272.5 ± 545.3	<0.001

*BMI* body mass index; *C/S* caesarean section.

^a^Continuous variables were presented as mean ± SD; qualitative variables were presented as *N* (%)

### Trends in maternal obesity and associated risks of adverse maternal and perinatal outcomes

**Fig. 2:**
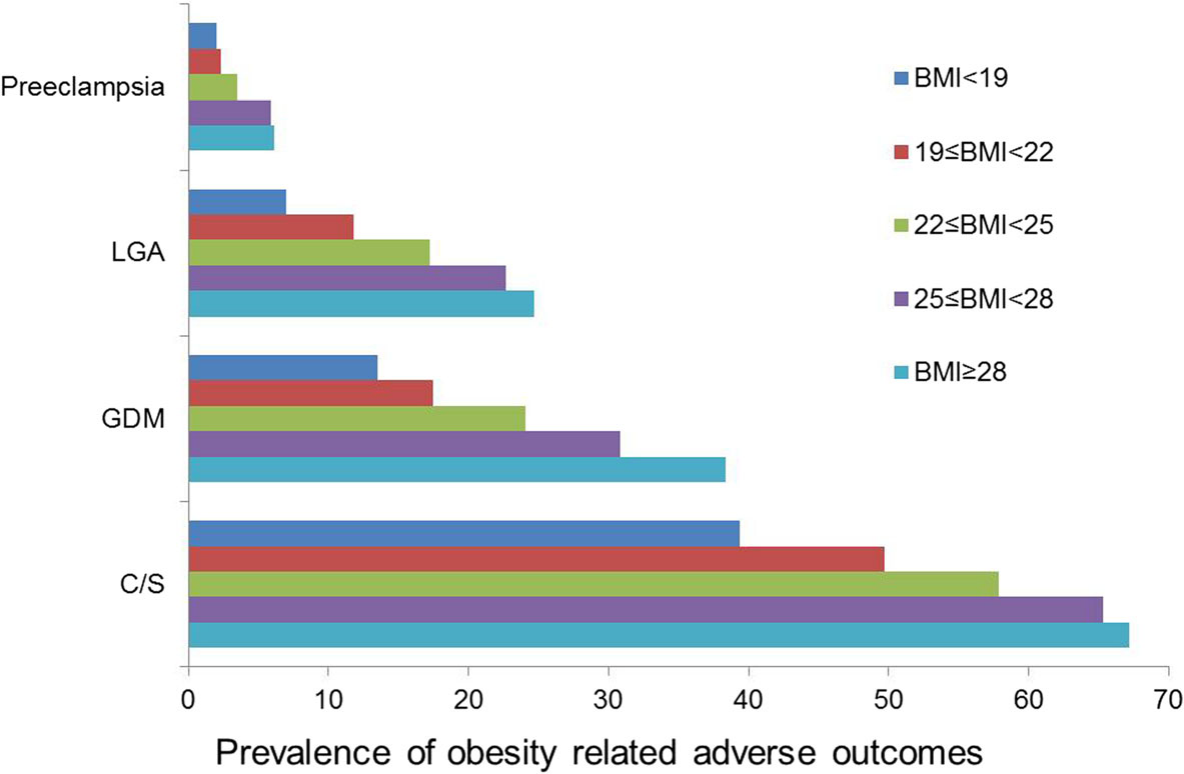
Prevalence of adverse pregnancy and birth outcomes by cumulative BMI. BMI: body mass index; GDM: gestational diabetes mellitus; C/S: caesarean section; LGA: large for gestational age

As presented in Table 2, with increasing BMI, the risk of GDM, preeclampsia, LGA, and C/S increased, indicating that women with obesity are at increased risks of the above complications. The prevalence of GDM, preeclampsia, C/S, and LGA manifested a positive linear trend with increasing BMI (P for trend < 0.001). In contrast, risks for SGA and OVD decreased with increasing BMI. Increasing BMI was not associated with an increased risk of PPH, preterm birth, shoulder dystocia, low Apgar’s score or admission to the NICU. Thus, we considered GDM, preeclampsia, C/S, and LGA as obesity-related adverse maternal and perinatal outcomes (Fig. 2).

**Table 2:** Maternal and neonatal complications of singleton pregnancies among women by pre-pregnancy obesity

Outcomes	BMI<19(kg/m^2^)		19≤BMI<22(kg/m^2^)		22≤BMI<25(kg/m^2^)		25≤BMI<28(kg/m^2^)		BMI≥28(kg/m^2^)		P
	*N* (%)	OR (95% CI)^a^	*N* (%)	Reference group	*N* (%)	OR (95% CI)^a^	*N* (%)	OR (95% CI)^a^	*N* (%)	OR (95% CI)^a^	for trend
Maternal complications											
GDM	424 (13.5)	0.87 (0.77-0.99)	896 (17.5)	1	584 (24.1)	1.34 (1.20-1.52)	193 (30.8)	1.82 (1.51-2.19)	76 (38.4)	2.73 (2.02-3.69)	<0.001
Preeclampsia	62 (2.0)	0.85 (0.62-1.56)	119 (2.3)	1	85 (3.5)	1.53 (1.15-2.03)	37 (5.9)	2.64 (1.81-3.85)	12 (6.1)	2.71 (1.47-4.99)	<0.001
PPH	163 (5.2)	0.73 (0.60-0.88)	355 (6.9)	1	161 (6.6)	0.97 (0.80-1.17)	52 (8.3)	1.25 (0.92-1.69)	12 (6.1)	0.88 (0.49-1.60)	0.008
Delivery mode											
OVD	293 (9.3)	1.28 (1.08-1.50)	350 (6.8)	1	152 (6.3)	0.99 (0.81-1.20)	24 (3.8)	0.63 (0.41-0.96)	2(1.0)	0.15 (0.04-0.62)	<0.001
C/S	1235 (39.4)	0.77 (0.70-0.85)	2543 (49.7)	1	1403 (57.9)	1.24 (1.12-1.38)	409 (65.3)	1.56 (1.29-1.88)	133 (67.2)	1.88 (1.36-2.59)	<0.001
Neonatal complications											
Preterm birth	222 (7.1)	1.02 (0.86-1.22)	366 (7.2)	1	216 (8.9)	1.24 (1.04-1.47)	58 (9.3)	1.25 (0.93-1.67)	22 (11.1)	1.56 (0.99-2.46)	0.001
LGA	220 (7.0)	057 (0.48-0.67)	605 (11.8)	1	416 (17.2)	1.53 (1.34-1.75)	142 (22.7)	2.14 (1.74-2.63)	49 (24.7)	2.42 (1.73-3.38)	<0.001
SGA	260 (8.3)	1.47 (1.23-1.75)	291 (5.7)	1	104 (4.3)	0.76 (0.60-0.95)	18 (2.9)	0.51 (0.32-0.83)	6 (3.0)	0.53 (0.23-1.21)	<0.001
Shoulder dystocia	1 (0.0)	0.06 (0.01-0.42)	27 (0.5)	1	7 (0.3)	0.58 (0.25-1.33)	2 (0.3)	0.68 (0.16-2.87)	1 (0.5)	1.04 (0.14-7.69)	0.102
Low Apgar’s score	106 (3.4)	1.09 (0.85-1.41)	152 (3.0)	1	82 (3.4)	1.18 (0.90-1.55)	16 (2.6)	0.90 (0.53-1.51)	12 (6.1)	2.17 (1.18-3.98)	0.656
Admission to NICU	665 (21.2)	0.99 (0.89-1.11)	1056 (20.7)	1	520 (21.5)	1.08 (0.96-1.22)	146 (23.3)	1.23 (1.01-1.50)	50 (25.3)	1.34 (0.97-1.86)	0.159

*BMI* body mass index; *GDM* gestational diabetes mellitus; *PPH* Postpartum hemorrhage; *OVD* Operative vaginal delivery; *C/S* caesarean section; *LGA* large for gestational age; *SGA* small for gestational age; *NICU* neonatal intensive care unit; *OR* Odds ratio; *CI* confidence interval

^a^Data adjusted for maternal age and previous caesarean section.

### Variable BMI group and obesity related adverse perinatal complications.

Table 3 showed the odds ratios associated with obesity-related adverse maternal and perinatal outcomes in the group of BMI ≥ 28kg/m^2^ in comparison with the group of 25 ≤ BMI < 28kg/m^2^. The risks of preeclampsia, C/S, and LGA were similar between the two groups, except that the risk of GDM was a little higher in the group of BMI ≥ 28kg/m^2^ (AOR was 1.47[95% CI: 1.05~2.07]).

**Table 3:** Variable BMI group and obesity related adverse perinatal complications^a^

	25≤BMI<28 (kg/m^2^)	BMI≥28(kg/m^2^)	
	Reference group	OR (95% CI)	AOR (95% CI) ^a^
GDM	1	1.40 (1.00-1.95)	1.47 (1.05-2.07)
Preeclampsia	1	1.03 (0.53-2.01)	1.03 (0.53-2.01)
C/S	1	1.09 (0.77-1.52)	1.20 (0.83-1.72)
LGA	1	1.12 (0.77-1.63)	1.12 (0.77-1.63)

*BMI* body mass index; *GDM* gestational diabetes mellitus; *C/S* caesarean section; *LGA* large for gestational age; *OR* Odds ratio; *CI* confidence interval.

^a^Data adjusted for maternal age and previous caesarean section.

### Effectiveness of the two obesity criteria to predict adverse outcomes

The sensitivities, specificities and Youden indexes of BMI ≥ 25 kg/m^2^ and BMI ≥ 28 kg/m^2^ in predicting adverse outcomes were shown in Table 4. Compare to BMI ≥ 28 kg/m^2^, the sensitivity of BMI ≥ 25 kg/m^2^ rose by 3-4 folds, while the decrease in specificity was minor. The Youden index of BMI ≥ 25 kg/m^2^ predicting the risk of GDM was higher than that of BMI ≥ 28kg/m^2^. Similar patterns were also detected for preeclampsia, C/S, and LGA. Finally, to predict the risk of the obesity-related adverse maternal and perinatal outcomes, we analyzed the Youden indexes of BMI ≥ 25 kg/m^2^ and BMI ≥ 28 kg/m^2^. The Youden index of BMI ≥ 25 kg/m^2^ was higher than BMI ≥ 28 kg/m^2^ (the difference of the Youden indexes was 0.065 [95% CI:0.051~0.079]), indicating that the former better identifies women at risk of obesity-related adverse maternal and perinatal outcomes.

**Table 4:** Sensitivities, specificities and Youden indexes of the two obesity criteria in predicting the risk of adverse outcomes

	Sensitivity (%)		Specificity (%)		Youden index (95% CI)		Difference of Youden index (95% CI)
	BMI_25_	BMI_28_	BMI_25_	BMI_28_	BMI_25_	BMI_28_	
GDM	12.4	3.5	94.0	98.7	0.06 (0.05~0.08)	0.02 (0.01~0.03)	0.04 (0.03~0.06)^b^
Preeclampsia	15.6	3.81	93.1	98.3	0.09 (0.05~0.13)	0.02 (0.01~0.04)	0.07 (0.03~0.10)^b^
C/S	9.47	2.32	95.1	98.9	0.05 (0.04~0.06)	0.01 (0.01~0.02)	0.03 (0.03~0.04)^b^
LGA	13.3	3.42	93.7	98.5	0.07 (0.05~0.09)	0.02 (0.01~0.03)	0.05 (0.04~0.07)^b^
Obesity related outcomes^a^	14.5	3.82	94.6	98.8	0.09 (0.08~0.11)	0.03 (0.02~0.03)	0.07 (0.05~0.08)^b^

*BMI* body mass index; *GDM* gestational diabetes mellitus; *C/S* caesarean section; *LGA* large for gestational age;

*CI* confidence interval.

^a^Obesity related outcomes required at least one in the following items: GDM, preeclampsia, C/S, and LGA

^b^The difference of Youden index between the two groups was statistically significant

## Discussion

In our study, women with obesity were at significantly increased risk of maternal and perianal complications. Although women whose BMI ≥ 28 kg/m^2^ were at high risk of complications, it was similar to those whose BMIs were between 25 and 28kg/m^2^. We found that BMI ≥ 25kg/m^2^ is better at predicting the risk of maternal and perianal complications than BMI ≥ 28kg/m^2^. Therefore, we suggested that BMI ≥ 25 kg/m^2^ is more appropriate than BMI ≥ 28 kg/m^2^ for defining obesity for pregnant women in South China.

BMI is a single acceptable predictor of adverse outcomes [18]. Our results show that increasing BMI is associated with increased risks of GDM, preeclampsia, C/S, and LGA, supporting results of previous studies [3–5, 19, 20]. SGA and operative vaginal delivery were negatively associated with increasing BMI, indicating that obesity was not associated with increased risk of SGA and operative vaginal delivery. Therefore, we considered GDM, preeclampsia, C/S, and LGA as obesity-related maternal and perinatal outcomes. Chui and colleagues concluded that a BMI of 25kg/m^2^ in Chinese adults was equivalent to a BMI of 30 kg/m^2^ in Caucasian subjects in terms of identifying those at risk of diabetes; [21] this finding indicates that the criterion of BMI should be race-specific. Leung et al. found that, compared with Caucasians, the impact of high BMI on gestational diabetes and preeclampsia in Chinese women was stronger [19]. However, the effect of different obesity criteria on predicting the risk of maternal and perinatal outcomes has not been addressed.

Youden’s index (also known as Youden’s J statistic) is a single statistic that captures the performance of a dichotomous diagnostic test. A higher value of the Youden’s index indicates better test authenticity. Aye M et al. used Youden’s index to identify the optimal cut-off point of BMI to predict the metabolic risk factors for metabolic syndrome among people aged 13–91 years [22]. Oliveira et al. used sensitivity, specificity, and overall accuracy (area under the curve) to describe the predictive performance of different diagnostic criteria of obesity as predictors of metabolic syndrome in adolescents [23]. In the research, we aimed to compare the WHO for Asians and WGOC obesity criteria’s effectiveness in predicting adverse maternal and perinatal outcomes, rather than finding the cut-point of the maximum predictive ability. Therefore, we use sensitivity, specificity and Youden index to evaluate the two cut-points’ predictive abilities. The sensitivity of BMI ≥ 25 kg/m^2^ was 3-4 folds higher than BMI ≥ 28 kg/m^2^, while the decrease in specificity was minor. The Youden index of BMI ≥ 25 kg/m^2^ was higher than that for BMI ≥ 28 kg/m^2^, indicating that BMI ≥ 25 kg/m^2^ has a better predictive value on the risk of obesity-related maternal and perinatal outcomes.

The association between the BMI and adverse maternal and perinatal outcome is likely to be driven by body fat. Although body fat was not measured in our study, many researchers have shown that BMI is an accurate assessment of the amount of adiposity. Chang et al. have revealed that Taiwan people with a BMI ≥ 25 kg/m^2^ had a similar body fat rate as Caucasians with BMI ≥ 30 kg/m^2^ [24]. Chen et al. have shown that, compared with the percent body fat obesity cut-off (≥ 40%), the BMI-obesity (BMI ≥ 25 kg/m^2^) criteria resulted in a better Youden index than the WGOC BMI criteria among middle-aged Chinese women [25]. In our study, the prevalence of obesity was 7.2% according to the WHO for the Asian BMI-obesity criterion; but was just 1.7% based on the WGOC criterion. This is because most of our subjects were from South China, which is a relatively slim population. However, if the WGOC standards (defining BMI ≥ 28 kg/m^2^ as obesity) are applied, a significant proportion of at-risk patients might be missed (about 5.5%). A lower BMI cut-off at 25 kg/m^2^ for defining obesity would better predict those at risk of adverse obstetric and perinatal outcomes and, therefore, enable the development of adequate support to reduce the incidence of adverse maternal and perinatal outcomes.

To our knowledge, this study is the first to compare the different obesity criteria’s effectiveness in predicting adverse maternal and perinatal outcomes. However, some limitations of our study should also be noted. Pre-pregnancy BMI was calculated using weight and height, and most of these data were likely to be self-reported. Nevertheless, self-reported BMI has been shown to have high specificity (96–98%) and sensitivity (86–92%) in women of childbearing age (20–49 years) [26]. Also, pre-pregnancy self-reported weight seems to be highly correlated with early pregnancy measured weight (*r* = 0.95) [27]. Also, this single-center study might not be representative of the general population. However, the center is the national key clinical department in South China with about 4000-delivery per year. Relevant guidelines were strictly followed in the management and care of pregnant women in this hospital, thereby avoiding the occurrence of adverse outcomes owing to the subjective factors of medical staff, which makes the outcomes more objective and reliable. In addition, the Youden indexes of BMI cut-offs for obesity were not high, because the sensitivities of them were low, which implicated that we couldn’t identify diseased and non-diseased individuals respectively only by BMI in clinical practice. We need to set up a model to predict a specific complication of pregnancy, in which more indicators should be included. These results could provide some evidence for further study. Lastly, lower BMI cut-off for obesity would cause a larger number of Chinese pregnant women to be treated as high-risk pregnancies. Therefore, a large, multi-center trial and further research addressing cost-effectiveness are warranted.

## Conclusions

Our results support the notion that obesity is associated with adverse perinatal outcomes. A lower BMI cut-off of 25 kg/m^2^ for defining obesity might be appropriate for Chinese pregnant women and help to identify those at risk of adverse maternal and perinatal outcomes. These results are important for women who are pregnant or are planning to become pregnant, as well as for clinicians who guide or provide prenatal counseling for women.

## References

[1] Collaborators GBDO, Afshin A, Forouzanfar MH, Reitsma MB, Sur P, Estep K, Lee A, Marczak L, Mokdad AH, Moradi-Lakeh M (2017). Health Effects of Overweight and Obesity in 195 Countries over 25 Years. N Engl J Med.

[2] Wang Z, Hao G, Wang X, Chen Z, Zhang L, Guo M, Tian Y, Shao L, Zhu M (2014). Current prevalence rates of overweight, obesity, central obesity, and related cardiovascular risk factors that clustered among middle-aged population of China. Zhonghua Liu Xing Bing Xue Za Zhi.

[3] Xiong C, Zhou A, Cao Z, Zhang Y, Qiu L, Yao C, Wang Y, Zhang B (2016). Association of pre-pregnancy body mass index, gestational weight gain with cesarean section in term deliveries of China. Sci Rep.

[4] Kim SS, Zhu Y, Grantz KL, Hinkle SN, Chen Z, Wallace ME, Smarr MM, Epps NM, Mendola P (2016). Obstetric and neonatal risks among obese women without chronic disease. Obstetrics and gynecology.

[5] Schummers L, Hutcheon JA, Bodnar LM, Lieberman E, Himes KP (2015). Risk of adverse pregnancy outcomes by prepregnancy body mass index: a population-based study to inform prepregnancy weight loss counseling. Obstet Gynecol.

[6] Liu Y, Dai W, Dai X, Li Z (2012). Prepregnancy body mass index and gestational weight gain with the outcome of pregnancy: a 13-year study of 292,568 cases in China. Arch Gynecol Obstet.

[7] Overcash RT, Lacoursiere DY (2014). The clinical approach to obesity in pregnancy. Clin Obstet Gynecol.

[8] Kominiarek MA, Gay F, Peacock N (2015). Obesity in Pregnancy: A Qualitative Approach to Inform an Intervention for Patients and Providers. Matern Child Health J.

[9] WHO. Obesity: preventing and managing the global epidemic. Report of a WHO Consultation. WHO Technical Report Series 894. Geneva: World Health Organization; 2000. 11234459

[10] Carpenter CL, Yan E, Chen S, Hong K, Arechiga A, Kim WS, Deng M, Li Z, Heber D. Body fat and body-mass index among a multiethnic sample of college-age men and women. J Obesity. 2013;2013. 10.1155/2013/790654 PMC3649342 23691288

[11] Shaikh S, Jones-Smith J, Schulze K, Ali H, Christian P, Shamim AA, Mehra S, Labrique A, Klemm R, Wu L. Excessive adiposity at low BMI levels among women in rural Bangladesh. J Nutr Sci. 2016;5. 10.1017/jns.2015.32 PMC4791523 27313847

[12] Choo V (2002). WHO reassesses appropriate body-mass index for Asian populations. Lancet.

[13] WHO EC (2004). Appropriate body-mass index for Asian populations and its implications for policy and intervention strategies. Lancet.

[14] Zhou B (2002). Coorperative Meta-Analysis Group Of China Obesity Task F: [Predictive values of body mass index and waist circumference to risk factors of related diseases in Chinese adult population]. Zhonghua Liu Xing Bing Xue Za Zhi.

[15] Zhou BF (2002). Cooperative Meta-Analysis Group of the Working Group on Obesity in C: Predictive values of body mass index and waist circumference for risk factors of certain related diseases in Chinese adults--study on optimal cut-off points of body mass index and waist circumference in Chinese adults. Biomed Environ Sci.

[16] American Diabetes A (2011). Standards of medical care in diabetes--2011. Diabetes Care.

[17] Gong XM, Li ZH, Yu RJ: Maternal and fetal general parameters. In: W.Y. Zhang, eds. Chinese Perinatology. Beijing: People’s Medical Publishing House; 2012. pp. 1592–1593.

[18] Bryant M, Santorelli G, Lawlor DA, Farrar D, Tuffnell D, Bhopal R, Wright J (2014). A comparison of South Asian specific and established BMI thresholds for determining obesity prevalence in pregnancy and predicting pregnancy complications: findings from the Born in Bradford cohort. Int J Obes (Lond).

[19] Leung TY, Leung TN, Sahota DS, Chan OK, Chan LW, Fung TY, Lau TK (2008). Trends in maternal obesity and associated risks of adverse pregnancy outcomes in a population of Chinese women. Bjog.

[20] Scott-Pillai R, Spence D, Cardwell CR, Hunter A, Holmes VA (2013). The impact of body mass index on maternal and neonatal outcomes: a retrospective study in a UK obstetric population, 2004-2011. Bjog.

[21] Chiu M, Austin PC, Manuel DG, Shah BR, Tu JV (2011). Deriving ethnic-specific BMI cutoff points for assessing diabetes risk. Diabetes Care.

[22] Mra A, Malek S (2012). Waist circumference and BMI cut-off points to predict risk factors for metabolic syndrome among outpatients in a district hospital. Singapore Med J.

[23] Oliveira RG, Guedes DP (2017). Performance of different diagnostic criteria of overweight and obesity as predictors of metabolic syndrome in adolescents. J Pediatr (Rio J).

[24] Chang CJ, Wu CH, Chang CS, Yao WJ, Yang YC, Wu JS, Lu FH (2003). Low body mass index but high percent body fat in Taiwanese subjects: implications of obesity cutoffs. Int J Obes Relat Metab Disord.

[25] Chen YM, Ho SC, Lam SS, Chan SS (2006). Validity of body mass index and waist circumference in the classification of obesity as compared to percent body fat in Chinese middle-aged women. Int J Obes (Lond).

[26] Kuczmarski MF, Kuczmarski RJ, Najjar M (2001). Effects of age on validity of self-reported height, weight, and body mass index: findings from the Third National Health and Nutrition Examination Survey, 1988-1994. J Am Diet Assoc.

[27] Park S, Sappenfield WM, Bish C, Bensyl DM, Goodman D, Menges J (2011). Reliability and validity of birth certificate prepregnancy weight and height among women enrolled in prenatal WIC program: Florida, 2005. Matern Child Health J.

